# Acute organ injury is associated with alterations in the cell-free plasma transcriptome

**DOI:** 10.1186/2197-425X-2-7

**Published:** 2014-02-21

**Authors:** John H Boyd, Melissa McConechy, Keith R Walley

**Affiliations:** Centre for Heart Lung Innovation, St. Paul’s Hospital, Room 166, 1081 Burrard Street, Vancouver, British Columbia V6Z 1Y6 Canada; University of British Columbia, Vancouver, British Columbia V6T 1Z4 Canada

## Abstract

**Background:**

Despite a genomic revolution in biological sciences, clinical medicine has yet to integrate diagnostics based upon gene expression into practice. While commonly used plasma protein assays rely on organ-specific origins, nearly all nucleic acid in whole blood is derived from white blood cells limiting their utility to diagnose non-immune disorders. The aim of the study was to use cell-free plasma to define circulating messenger RNA sequences diagnostic of acute organ injury, including myocardial infarction (MI) and acute kidney injury (AKI).

**Methods:**

In healthy human subjects (*N* = 4) and patients with acute MI (*N* = 4), we characterized the concentration and nature of circulating plasma RNA through spectrophotometry and chromatography. Through reverse transcriptase polymerase chain reaction (RT-PCR) of amplicons up to 939 base pairs, we determined whether this mRNA was intact but of insufficient quantity to sequence. In mice, we induced an acute anterior myocardial infarction through 1 h of ischemia followed by reperfusion of the left anterior descending (LAD) artery. We compared the cell-free plasma transcriptome using cDNA microarray in sham-operated mice compared to ischemia upon reperfusion and at 1 and 4 h. To determine organ specificity, we compared this profile to acute ischemia-reperfusion of the kidney.

**Results:**

In humans, there is more plasma RNA in those with acute MI than in healthy controls. In mice, ischemia-reperfusion of the LAD artery resulted in a time-dependent regulation of 589 circulating mRNA transcripts with less than a 5% overlap in sequences from acute ischemia-reperfusion injury of the kidney.

**Conclusions:**

The mRNA derived from cell-free plasma defines organ injury in a time and injury-specific pattern.

**Electronic supplementary material:**

The online version of this article (doi:10.1186/2197-425X-2-7) contains supplementary material, which is available to authorized users.

## Background

Microparticles are released by activated, damaged, apoptotic, and necrotic cells into the circulation [1–4]. In health, microparticles are most commonly platelet-derived, and a class of procoagulant apoptosis-marker positive (annexin V) microparticles is also found [1]. In a number of vascular disease states, endothelial-derived microparticles become important [1]. The cellular source of microparticles can be determined by examining the expression of cell-surface molecules. For example, platelet-derived microparticles are identified by the expression of CD41 and endothelial-derived microparticles are identified by their expression of CD146 [1]. Importantly, microparticles contain nucleic acids from their cells of origin [5, 6]. Plasma-derived microparticle mRNA has been used to distinguish patients who have colon cancer from those free of the disease [5]. Conceivably, the sequence and copy number of expressed genes (mRNA) derived from cell-free plasma could function as cardiac-specific biomarkers in diseases such as ischemia-reperfusion and acute heart failure. In a more limited (fewer sequences) way, this has recently been found to be the case for miRNA contained in microparticles following ischemic cardiac injury [7]. Of great interest to clinicians and scientists, analysis of the circulating transcriptome following injury could identify important molecules or pathways which are regulated as a result of the insult. Through the use of the cell-free component of blood, we can eliminate the leukocyte-derived transcriptome, which to date has limited the utility of organ-specific nucleic acid biomarkers.

Accordingly, we characterized plasma-derived mRNA with respect to concentration, intactness, and chromatographic characteristics in humans. Next, we measured mRNA expression patterns using DNA microarray over time in the cell-free fraction of blood samples in mice after one hour ischemia followed by reperfusion of the left anterior descending (LAD) artery. To determine whether these sequences were specific to cardiac injury, we compared them to both sham-operated mice and to unilateral ischemia-reperfusion of the kidney. We found that plasma-derived mRNA exhibits an organ and time-dependent signature following ischemic injury.

## Methods

### Human studies

#### Blood samples

International review board (IRB) approval was obtained for this study from the Providence Health Care IRB; written informed consent was obtained from all subjects. Whole blood was collected from healthy control subjects and patients at primary angiography for acute myocardial infarction (MI) in 5-mL EDTA blood collection tubes. The blood was centrifuged at 1,000 × *g* for 10 min. Plasma (250 μL) was mixed with 750 μL TRIzol LS (Invitrogen, Carlsbad, CA, USA) and was stored at -80°C. RNA was extracted as per the manufacturer's instructions, cleaned on a minispin column (Qiagen, Venlo, The Netherlands), and was treated with DNAse (Ambion, Austin, TX, USA). A spectrophotometer (Nanodrop, Wilmington, DE, USA) was used to quantitate RNA for all samples, and chromatographic characteristics were assessed using Agilent 2100 Bioanalyzer (Agilent, Santa Clara, CA, USA).

#### RNA stability

Blood was drawn from healthy subjects in EDTA collection tubes (as above) and cooled to 4°C. In the first set of experiments RNA was (1) immediately extracted, (2) extracted after 6 h, or (3) extracted after 24 h. Total RNA was then measured. In subsequent experiments, blood was immediately centrifuged at 4°C, and plasma was mixed with TRIzol LS and stored at -80°C.

#### RNA integrity

The Invitrogen One-Step SYBR Green RT-PCR (Invitrogen) kit components were used for the PCRs. Plasma RNA was used as a template for amplification of three different sizes of amplicons for GAPDH (107, 506, and 939 bp) to assess the integrity of the extracted RNA. DNase-treated RNA (100 ng) was added as template to SuperScript One-Step RT-PCR using Platinum Taq protocols to yield the corresponding cDNA. RT-PCR conditions were 50°C for 30 min, 94°C for 2 min, followed by 40 cycles of 94°C for 15 s, 58°C for 30 s, and 72°C for 2 min, which was followed by 72°C for 5 min and 4°C as the final temperature. To analyze the final amplicons, 9 μL of each PCR product was loaded into each lane of 1% agarose gel.

#### Linear amplification of cDNA

We used the SMARTer PCR cDNA synthesis protocol (Clontech, Mountain View, CA, USA) to first generate cDNA and then amplify the cDNA with 40 cycles of thermal cycling. To visualize the cDNA bands, 9 μL of the product was run on 1% agarose gel.

### Murine studies

All animal studies were approved by the University of British Columbia animal ethics committee.

#### Ischemia-reperfusion model

We chose ischemia-reperfusion (I/R) as a model of organ injury. Cardiac I/R injury was performed as we have previously published [8]. Briefly, while intubated and ventilated, the proximal LAD artery was clamped for 1 h followed by reperfusion. Ischemic myocardial injury was verified using ECG monitoring. For kidney I/R injury in separate mice, following midline laparotomy, renal arterial blood flow was interrupted for 1 h followed by reperfusion. In each model, separate mice were sacrificed in a time sequence. The time point immediately following reperfusion was *T* = 0; 1 h after reperfusion, *T* = 1; and 4 h after reperfusion, *T* = 4. Both normal mice (control) and sham-operated mice (SHAM) for each procedure served as controls (*N* = 3 per condition and time).

#### Expression array measurements

The goal of these experiments was to determine whether Affymetrix small-sample preparation could generate sufficient labeled RNA to successfully perform expression microarray analysis on plasma-derived mRNA. Plasma RNA was extracted using TRIzol LS followed by column purification. RNA was reverse-transcribed and hybridized as per the Affymetrix gene 1.0 ST kit instructions (Affymetrix, Santa Clara, CA, USA).

#### Linear amplification of mRNA

The SMART mRNA (Clontech) protocol was carried out using PCR Primer IIA to help with the amplification of the second strand cDNA, instead of just the extension. The cDNA had gone through 40 cycles as previously performed using the SMARTer PCR cDNA synthesis protocol, and 9 μL of the product was analyzed on 1% agarose gel. For subsequent expression array hybridization, a minimum of 200 ng/μL final concentration of labeled RNA was used.

#### Statistical analysis

Microarray data was normalized using Flexarray (Genome Quebec, Montreal, Canada) robust spline RMA, and groups were compared using a *t* test. Significance was set at *p* < 0.05, and biological significance was set at an absolute fold change of >2. Pathway analysis was performed using Ingenuity Pathway Analyzer (Ingenuity, Redwood City, CA, USA).

## Results and discussion

### Results

**Figure 1:**
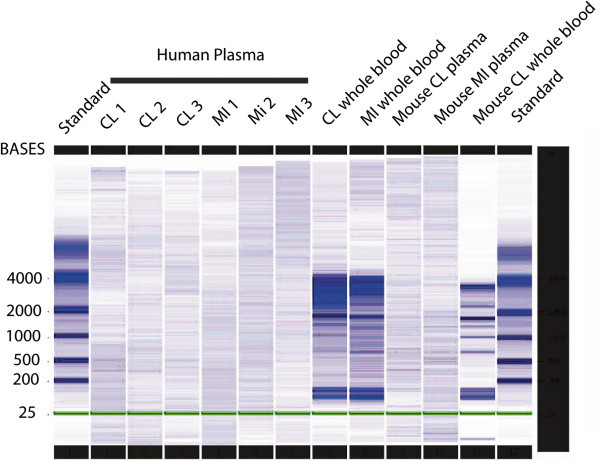
**Plasma drawn from human controls and MI patients and from control and MI mice.** It was analyzed using Agilent Bioanalyzer. On each end, there are standard RNA ladders. Plasma-derived RNA does not contain the dense ribosomal bands seen just below the 4,000 and 2,000 BASE standards (28S and 18S respectively) with whole blood RNA extraction.

**Figure 2:**
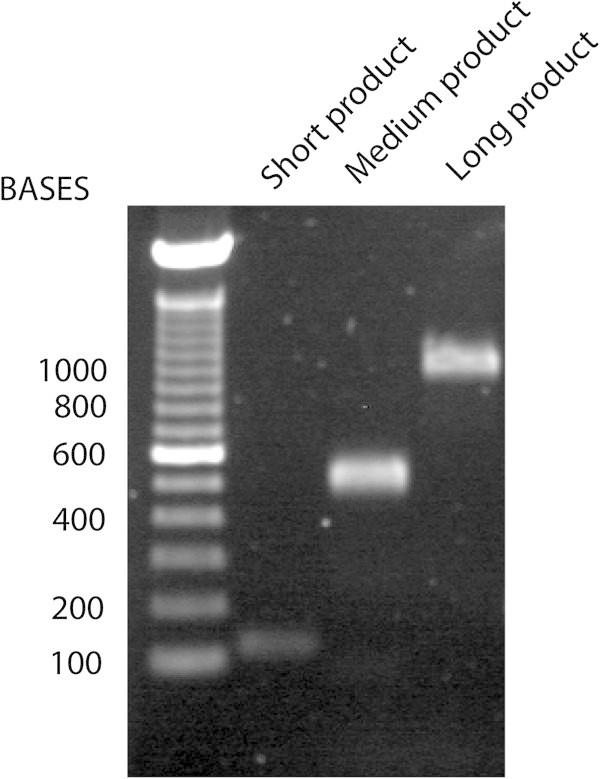
**Following RT of RNA-derived from human plasma, PCR was performed for increasing amplicon lengths of GAPDH.** This was done to determine the integrity of the RNA. Lane 1 has a faint amplicon of 107 bp, lane 2 has an amplicon of 506 bp, and lane 3 has the largest amplicon at 939 bp.

*In human subjects, RNA that contains intact species was increased in patients with acute MI who were relatively stable for over 24 h*. The final concentration of plasma RNA obtained from control subjects (*N* = 4) was 1.9 ± 0.1 μg/mL blood, while in patients with acute MI (*N* = 4) who had plasma drawn at primary angiography, the mean concentration of plasma RNA was 6.5 ± 2.4 μg/mL blood. In two control subjects, the stability of RNA was determined over 24 h. At 6 and 24 h, the mean concentration decreased to 44 ± 24% and 40 ± 21% of baseline values, respectively. In contrast to RNA derived from whole blood, plasma-derived RNA does not contain ribosomal bands (Figure 1) but does contain intact mRNA species as we were able to amplify GAPDH products sequentially up to 939 bp (Figure 2).

**Figure 3:**
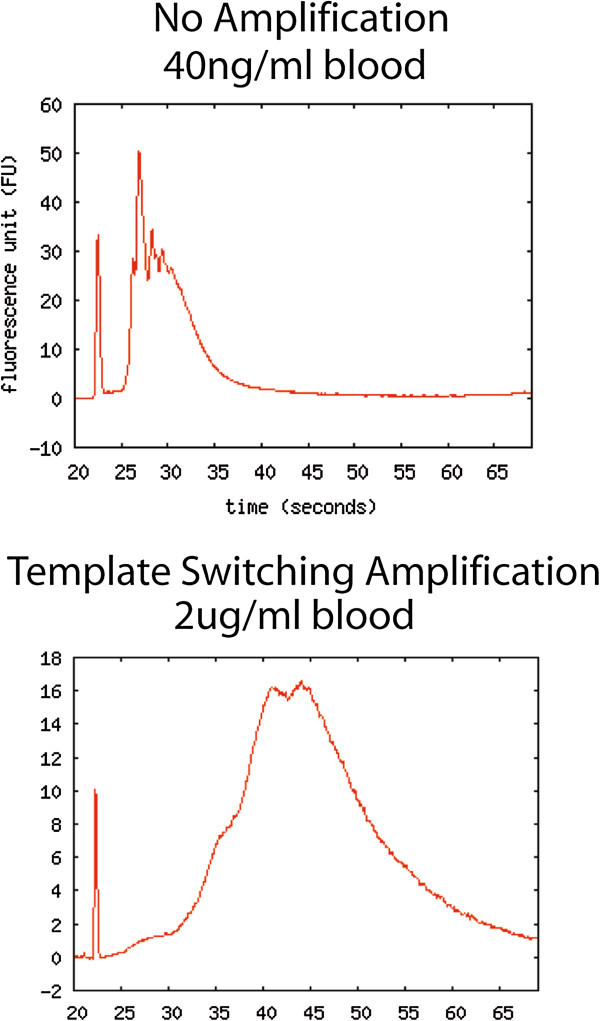
**Representative fluorescently labeled cDNA prepared using the Affymetrix small-sample method.** It is run on an Agilent 2100 Bioanalyzer both with and without previous template switching amplifications. Amplification results in a greater area under the curve, representing the mass of incorporated cDNA (2 μg), are compared to only 40 ng in an unamplified sample. The peak also shifts right with amplification, indicating larger cDNA sequences.

*Phenol extraction followed by column purification was able to extract enough polyA tailed mRNA for three to five PCRs, but intact mRNA species were not abundant enough to allow DNA microarray. Modified template switching amplification allowed us to perform DNA microarray.* Usual Affymetrix small-mass RNA amplification to incorporate fluorescent probe into the plasma-derived RNA for subsequent Affymetrix Gene 1.0 ST microarray was performed by reverse transcription followed by two rounds of *in vitro* transcription with T7 RNA polymerase and final regeneration of fluorescently labeled cDNA. The quality of fluorescently labeled cDNA was analyzed using the Agilent 2100 Bioanalyzer (Figure 3, upper panel). This process was unable to yield adequate labeled cDNA for microarray, averaging 40 ± 3.5 ng in total per sample (*N* = 13). Using modified template switching resulted in a tremendous increase in useable labeled cDNA to 2.0 ± 0.12 μg per sample (*N* = 30; Figure 3, bottom panel).

**Figure 4:**
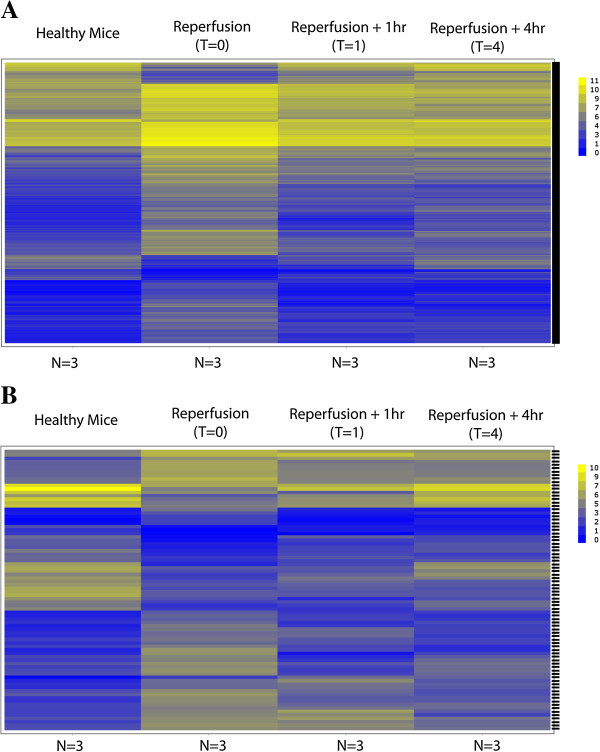
**Heat maps of differentially expressed genes comparing ischemia-reperfusion to sham operation (group mean data).** They visually demonstrate significantly regulated genes upon reperfusion (*T* = 0). Mouse **(A)** MI and **(B)** KI. Significant regulation at *T* = 0 was defined as *p* < 0.05 and an absolute fold change ≥ 2. Gene expression is plotted with each gene as a unique horizontal bar and is expressed as log_2_; therefore, compared to sham, a fold change of 0.5 represents a downregulated expression at an absolute fold change of -2. The gene expression intensity legend is located in the upper right of each panel. Gene expression is followed at 1 and 4 h following reperfusion. In A (MI), there are 589 genes depicted in the heat map, while in B (KI), there are 83 genes which contain easily visualized blocks of regulated genes (both up and down) and identify the respective injuries.

*In a mouse model of ischemia-reperfusion of the LAD artery, there are time-dependent plasma-derived molecular signatures of MI, which are distinct from sham operation or acute kidney injury (KI).* Once we had proven that there was intact RNA in plasma and that we could amplify it to a degree which would allow genome-wide interrogation, we used mouse models of acute organ injury to identify differentially expressed genes following injury. Heat maps (Figure 4A,B) demonstrate significantly regulated genes detected with Affymetrix Gene 1.0 ST arrays at *T* = 0, 1, and 4 versus sham operation. Significant regulation was defined as a *p* value that is <0.05 and a fold change that is ≥2. The heat maps demonstrate organ and time specificity. Immediately upon reperfusion of the LAD artery (*T* = 0), the largest regulation of plasma-derived genes was observed, with 589 significantly regulated versus sham operation. This number decreased to 63 genes at 1 h and 93 genes at 4 h. To identify whether circulating genes were increased non-specifically during organ injury or conversely were specific for the heart, we used a model of kidney ischemia-reperfusion injury as a comparator. Table 1 depicts overlapping genes. At comparable times following injury, there is only a 0.5% overlap between the genes found circulating as a result of kidney ischemia-reperfusion injury and those in myocardial ischemia-reperfusion injury. Altogether, over the 4-h period, there is a 4% overlap in differentially expressed genes comparing MI to KI. The complete gene lists with fold change and *p* values are found in Additional file 1.

**Table 1:** **Overlap of genes between MI and KI**

	MI***T*** = 0	MI***T*** = 1 h	MI***T*** = 4 h
	(589 genes)	(63 genes)	(83 genes)
**KI** ***T*** **= 0 (83 genes)**	**3**	*2*	*1*
**KI** ***T*** **= 1 h (243 genes)**	*27*	**0**	*2*
**KI** ***T*** **= 4 h (30 genes)**	*2*	*0*	**1**

Each organ injury was compared with its sham operation, and significant regulation of gene expression was defined by a *p* value that is <0.05 and biological significance set at an absolute fold change ≥2. At the same time following ischemic injury (bold), there is a maximum overlap of 0.5%. Altogether, there is a 4% overlap between MI and KI during the first 4 h of reperfusion.

#### Transcriptome

In the MI model, we then used Ingenuity's (Qiagen) pathway analyzer to determine the top canonical pathways for differentially expressed genes. There were four significantly regulated pathways: (1) oxidative phosphorylation (15/166 genes, *p* value = 7^-10^), (2) mitochondrial dysfunction (11/172 genes, *p* value = 2^-6^), (3) ubiqionone biosynthesis (7/119 genes, *p* value = 2^-5^), and (4) inositol metabolism (4/98 genes, *p* value = 0.001).

### Discussion

The pattern of expressed mRNA, the ‘transcriptome’, is specific depending upon the cell type and changes over time, reflecting the physiologic state of the cell. The enormous advantage an mRNA diagnostic would enjoy over protein is that unlike the lengthy optimization required for each protein assay, the ability to detect newly discovered mRNA sequences is as easy as synthesizing complementary probes. This can be accomplished in a few hours. Furthermore, on numerous detection platforms (for example RT-PCR, microarray, bead assays), measurement of mRNA sequences can be easily multiplexed. This could greatly increase the sensitivity and specificity of a diagnostic test using a panel of dozens or even hundreds of mRNA sequences rather than the current system whereby clinicians base decisions on a single protein level. Cell-free plasma DNA, elevated in numerous acute disease processes, has been proposed as a biomarker of organ injury [9–11]. However, unlike the expressed genome (mRNA), DNA reflects the genes one is born with rather than acute changes in physiology. Though there is a theoretical advantage to mRNA-based diagnostics, transcriptome measurement has had little impact on clinical medicine because it is extremely difficult to access useful mRNA. It is not feasible to biopsy the diseased heart or lung to isolate mRNA biomarkers. Within a clinical setting, mRNA must instead be assayed from the blood. While blood is an abundant source of RNA, it is overwhelmingly derived from white blood cells. However, the cell-free portion of blood (plasma) contains submicron-sized particles containing mRNA derived from the tissue [5]. It is this mRNA fraction that we believe holds the key to merging genomics and diagnostics. If one could measure tissue-derived mRNA in blood, sequences could serve as both diagnostic biomarkers and a window into complex biology.

Human plasma is known to contain cell-free nucleic acids, although the nature and concentration of these molecules are not well understood. It is well established that plasma contains circulating nucleic acids; however, until very recently, they were assumed to be present at extremely low concentration and were highly degraded due to the presence of circulating ribonucleases [9]. It is now recognized that there is a relatively complex mixture of free nucleic acids in the plasma, including DNA [9, 11], fetal RNA in maternal plasma [12], and what is thought to be secreted RNA (RNA which is enriched in mRNA compared to the usual total cellular RNA) [5, 13]. Secreted RNA appears to be protected against enzymatic degradation because it is encapsulated in vesicle-like structures that are less than 1 μm in diameter [5]. These microparticles, because of the active nature of their contents, have been found to be biologically active, are procoagulant, and have atherogenic and immune-modulating effects [7, 14–20]. While exogenous or ‘spiked’ mRNA added to plasma is immediately degraded, the endogenous mRNA is protected and thus relatively stable [5, 21]. We found significant amounts of RNA persisting in the cell-free fraction of whole blood for over 24 h when cooled to 4°C. As we have shown in Figure 2, secreted RNA lacks dense ribosomal bands usually associated with intact cellular-derived RNA. This finding has also been reported by other investigators [11, 21]. It is believed that vesicles are actively secreted and that messenger RNA is packaged preferentially over ribosomal RNA [9, 22]. The evolutionary reasons for this secretion of expressed genes are not currently known. Despite this, we have been able to amplify sequences of nearly 1,000 base pairs using RT-PCR, indicating that at least some of this mRNA are intact.

Not only is the cell-free plasma RNA concentration increased in acute illness, there are disease- and time-specific regulations of expressed mRNA sequences. We compared the circulating RNA levels in five patients with early ST elevation myocardial infarction (MI) with age-matched healthy controls. We found that serum RNA levels were increased over threefold in MI patients than in healthy controls, suggesting that acute illness increases circulating RNA levels. When plasma mRNA is purified and extracted by phenol and/or microspin columns, the mass of intact polyA mRNA in the total mRNA mixture is too low (in picograms rather than the necessary 1 to 10 μg) to be characterized using direct sequencing or microarray. To surmount this problem in a cost-effective manner, we linearly amplified all mRNA species by adapting template switching PCR, allowing us to extract 5 to 10 μg of amplified polyA-tailed mRNA from 1 ml of blood, sufficient for downstream sequencing or microarray analysis.

Once we found that there were indeed intact circulating mRNA species in plasma, we went on to establish that organ injury is associated with unique transcriptome profiles. We used separate mouse models of acute myocardial ischemia-reperfusion injury and renal ischemia-reperfusion injury. Our goals were to (1) identify sequences which could uniquely identify cardiac or renal injury (crucial to a sensitive diagnostic) and (2) to establish the degree that sequences overlapped between organ injuries (low overlap as key to diagnostic specificity). We chose to examine very early time points, a time in which the need for novel diagnostics exists as it is well before traditional protein assays such as troponin are generally elevated in MI. By sampling plasma at three time points (immediately following reperfusion, 1 h post reperfusion, and 4 h post reperfusion), we found differentially expressed genes (589 in the mouse model of MI and 243 in renal injury; *p* < 0.05 and fold change > 2) defining both time-and organ-specific patterns of circulating mRNA species. Less than 5% of sequences were common to both cardiac and renal injuries, indicating high specificity. Pathway analysis of the regulated circulating genes revealed a stress response with oxidative phosphorylation and mitochondrial dysfunction, the two top canonical pathways.

*Limitations* to this study include the very small sample size (*N* = 8 in human RNA concentration and *N* = 3 in mouse studies). We feel that this report is hypothesis testing, and while observational in nature, it opens the door to what is possible with this, as yet, unexploited source of circulating mRNA. The regulated genes must be regarded as having the potential to diagnose acute myocardial infarction rather than representing a ready-made diagnostic panel. Further well-designed human studies are necessary for proper validation.

## Conclusions

Myocardial ischemia-reperfusion injury results in the release of mRNA into the systemic circulation. This transcriptome can be detected and interrogated from the cell-free fraction of peripheral blood samples. It appears to be informative regarding myocardial signaling pathway activity in this pathologic state.

## Electronic supplementary material

Additional file 1: **Complete gene lists with fold change and** ***p*** **values.** (XLSX 430 KB)
